# Behavioural and Physiological Changes in a Herd of Arabian Mares after the Separation of Individuals Differently Ranked within the Dominance Hierarchy

**DOI:** 10.3390/ani11092694

**Published:** 2021-09-14

**Authors:** Anna Stachurska, Anna Wiśniewska, Witold Kędzierski, Monika Różańska-Boczula, Iwona Janczarek

**Affiliations:** 1 Department of Horse Breeding and Use, Faculty of Animal Sciences and Bioeconomy, University of Life Sciences in Lublin, 20-950 Lublin, Poland; anna.stachurska@up.lublin.pl (A.S.); iwona.janczarek@up.lublin.pl (I.J.); 2 Department of Biochemistry, Faculty of Veterinary Medicine, University of Life Sciences in Lublin, 20-950 Lublin, Poland; witold.kedzierski@up.lublin.pl; 3 Department of Applied Mathematics and Computer Science, Faculty of Production Engineering, University of Life Sciences in Lublin, 20-950 Lublin, Poland; monika.boczula@up.lublin.pl

**Keywords:** horse, social herd, hierarchy, separation, behaviour, agitation

## Abstract

**Simple Summary:**

There are many situations in riding facilities and studs in which horses have to be separated out of a group, for example, when they are needed for a riding lesson, training purposes or veterinary treatments. Such a group may form a social herd in which a dominance hierarchy with dominant and submissive members is established. In the study, we investigated how mares respond to a short separation of some of the herd members and whether the response differs according to the rank order of the mares separated. The response was determined with rates of different behaviours showing agitation or calmness, times of locomotion and physiological parameters. The results of the experiment show that the separation of some mares increases the agitation in the remaining herd. In spite of the fact that such situations are constantly repeated in practice, they are stressful for the horses. The reaction of the remaining herd does not depend strictly on the composition of the mares separated regarding their rank in the dominance hierarchy, i.e., it does not differ consistently when a dominant, mixed or submissive group of mares is separated.

**Abstract:**

Horses in a herd develop and maintain a dominance hierarchy between all individuals. There are many situations in riding facilities and studs in which horses have to be separated out of a group. The aim of the study was to determine the rate of behaviours, level of locomotor activity and cardiac activity variables in a herd of horses during a short social separation of individuals differently ranked in the dominance hierarchy. Twelve adult Arabian mares were involved. A behavioural test had been performed before the main experiment to determine the rank order of the mares in this social herd. Three tests were performed when a dominant, mixed and submissive three-member group of mares was separated for 10 min. The response of the remaining herd was determined by a rate of behaviours, time of locomotor activity and cardiac parameters. The results of the experiment reveal evident changes towards emotional arousal in the social herd elicited by a short separation of some conspecifics. The herd created by humans preserves the sensitivity to a temporary loss of its members. The response of the remaining herd does not depend strictly on the composition of the separated mares regarding their rank in the dominance hierarchy.

## 1. Introduction

Social bonds are observed in many studies performed on feral and free-ranging horses slightly impeded by human interference e.g., [1,2,3,4]. The same social behaviour patterns are observed in outdoor-living or pastured horses under extensive human management when given the opportunity [5]. These observations show the natural propensity of horses to evolve long-term bonds within herds. Under natural conditions, a herd usually consists of a stallion, one or more, often a few, adult, usually unrelated mares and their foals [6]. The mares evolve affiliative social integration, which has strong benefits for them, for example, it enables allogrooming, reduces harassment by males and increases foal birth rates [1,4]. Both mares and foals prefer to bond with peers within their sex [7].

On the other hand, the horses in a herd develop and maintain the dominance hierarchy between each individual mainly through agonistic interactions. Most studies indicate that agonism is strongly correlated with the dominance rank among females [2,3,8]. The hierarchy is relatively stable and ranks positively with horses’ age, total aggressiveness, body size and condition, as well as the duration of residency in the herd, although those correlations are not found universally [5,8,9,10,11]. The horse’s sex is of great importance for the hierarchy. The stallion is not the highest-ranked horse, but a mare is the leader of a herd [2,3,12]. The reproductive status of females also influences their social dominance in a band [13]. The more dominant horses manifest aggression more often than individuals ranked lower in the hierarchy [8].

Groups of pastured horses under human management are usually maintained without a stallion. Horse owners tend to keep horses in homogenous groups to facilitate management and avoid injuries [14]. However, broodmares with their foals are sometimes maintained with barren mares and geldings [7]. Keeping breeding mares together with barren mares and adult geldings in a herd does not interfere with the normal species-specific behaviour [13,14]. The stability of group composition in pastured horses facilitates establishing the dominance hierarchy. In turn, the established hierarchy reduces aggression levels in a herd, which correlates with the welfare of horses. Unstable social grouping increases aggression and may lead to injury [12,13].

Social ties within a horse herd arise thanks to gregariousness, i.e., dependence on conspecifics in a herd, especially manifested by the reactivity to social isolation. Horses are gregarious animals, hence social isolation can elicit different responses in them, for example, vocalisation [15]. The dependence is opposite to independence and correlates with gregariousness and sociability. Independence means an individual’s ability to function without the social support of conspecifics [16]. The horses vary regarding the levels of these traits. Lower sociability indicates that some horses may be more self-reliant, relaxed and better able to cope with social isolation than their conspecifics. Independence and boldness are separate traits in horses, but only boldness increases with age [15,16]. The sociability depends on the breed, for example, Irish Draught and American Quarter horses rank lower for this trait than Arabians and Thoroughbreds [17]. Horses that do not cope well when separated from conspecifics, for example, Thoroughbreds, are at a high risk of stereotypic behaviours [18].

Some studies report the consequences of introducing unfamiliar newcomers into a social group of horses or the reaction of an individual being separated from such a group [15,19,20,21]. However, no information is available on the response of the herd to taking out a member. Hartmann et al. [22] suggested that investigations were warranted on the consequences of removing either higher or lower ranked horses from their group on collective movements. There are many situations in riding facilities and studs in which horses have to be separated out of a group, for example, when they are needed for a riding lesson, training purposes or veterinary treatments. It is interesting to investigate how the remaining group reacts, whether it notices the lack of some conspecifics at all or experiences a stress. We hypothesized that a short separation of some individuals from a herd elicits behavioural, locomotor and physiological changes in the remaining herd and those changes differ depending on the hierarchy rank of the conspecifics separated. The aim of the study was to determine the rate of behaviours, level of locomotor activity and cardiac activity variables in a herd of horses during a short social separation of individuals differently ranked in the dominance hierarchy.

## 2. Materials and Methods

### 2.1. Horses

In the study, 12 purebred Arabian mares aged five to ten years were involved. The mares had been kept in the breeding facility for at least one year. They were maintained in single box stalls. The construction of the stall’s side walls allowed eye contact between the horses, whereas the frontal walls made it possible for horses to touch other horses from neighbouring stalls. The mares were fed three times daily with a concentrate and meadow hay. The morning feeding was carried out at 6 a.m. The mares had stayed together in a paddock or pasture with access to water from 8 a.m. to 5 p.m. each day for at least the last six months. The pasture-beds for the whole group of mares were 3000–3500 m^2^ each. The foals of the mares were already weaned at the time of the experiment. Nine mares were in the second trimester of pregnancy and three mares were barren. 

### 2.2. Procedure of Testing Dominance Hierarchy

A behavioural test had been performed to determine the rank order of mares in this social herd before the experiment began. Accordingly, at 8 a.m. on three consecutive days, all the mares were turned out freely into a small paddock. A crib 100 cm long, 30 cm deep and 20 cm wide holding the concentrate was located there. The mares were familiarised with this crib. The rank in the hierarchy was determined according to the order of approaching the crib and beginning to consume the concentrate. Mares that began to eat simultaneously were considered to be of the same rank. Successive mares that completed the test (after eating a few bites of the concentrate) were taken away from the crib and tied to the fence of the paddock. The concentrate was added successively until the test was completed. The results of the test were similar on consecutive days. They allowed us to distinguish the following hierarchy:

- The leader of the herd always approached the crib and began to consume the concentrate first and alone;
- Two subdominant mares simultaneously began to consume the concentrate in second place;
- A group of seven mares ranked third;
- A subservient mare ranked fourth;
- A solitary mare, which usually kept away from the herd, then approached the crib a few minutes later and was ranked fifth.

### 2.3. Procedure of Main Tests

The experiment was carried out at 8:00 a.m., i.e., two hours after the morning feeding, on three days. Another test was performed on each experimental day. The experimental days were split up by three-day intervals to prevent the habituation of the mares to the experimental procedure. The mares were turned out into a paddock of 50 × 70 m size. Water and food were not available in the paddock and no green growth around the paddock was present. On each experimental day, a sport-tester (Polar ELECTRO OY-RS800CX sport140, Polar Electro Oy, Kempele, Finland) was attached to a belt placed around the chest of each horse and activated to measure the cardiac variables. The horse was then left in its box for 5 min to become accustomed to the device. The mares had already been familiarised with the device a week before the beginning of the experiment. 

Each test consisted of the following three 15 min phases:

- Phase 1 after releasing the mares into the paddock;
- Phase 2 when selected mares were separated from the herd;
- Phase 3 after bringing the separated mares back to the herd.

The separated mares were led by familiarised grooms to their own boxes in the stable. The cribs in the boxes contained some concentrate to calm the horses and occupy them with eating. After the second phase, the mares were led back to the herd. The tests differed with the composition of the groups of three mares separated:

- The first test carried out on the first experimental day—the group dominant over the rest of the herd including the leader and two subdominant mares;
- The second test carried out on the second experimental day—the mixed group including the leader, subservient and solitary mares;
- The third test carried out on the third experimental day—the submissive group including two subdominant mares simultaneously submissive to the leader and the solitary mare submissive to the whole herd. The measurements conducted in each phase concerned the herd without the mares separated in phase 2.

### 2.4. Measurements within Main Tests

The rate of behaviours, time of locomotor activity and cardiac parameters were recorded separately for each phase of the experiment. The following behaviours observed were assumed to display agitation: Vocalisation, defecation/urination, high head and tail positions, clinging to conspecifics, aggressive posture and ears pressed caudally or held forward (Table 1). Rest standing, drowsiness, play, mutual grooming and ears held laterally were considered to be signs of calmness [23,24,25,26,27]. The prevalence of the behaviours was recorded regardless of how long they lasted. The locomotor activity was considered regarding the time of a walk, trot, canter, jumps and single steps [28]. More than three steps were assumed to be a gait and fewer than three steps were classified into single steps. Increased locomotion activity was considered as a sign of agitation. The time of each gait repeated within a phase was totalled.

The heart rate (HR; beats per min) and the root mean square of the successive differences in beat-to-beat intervals (RMSSD; ms) were also analysed. The HR increases as a result of elevated sympathetic nervous system activity, whereas augmented RMSSD shows the predomination of parasympathetic nervous system activity. Hence, an increased HR was considered as a sign of agitation, whereas increased RMSSD was a sign of calmness.

### 2.5. Statistical Analysis

The statistical analysis was performed utilising STATISTICA 13.3 and R software (R version 4.0.3). The normality of the data distribution was assessed with the Shapiro–Wilk test. The non-parametric analysis of variance for repeated measures (Friedman’s test) was used since the distribution of the behavioural and locomotor data was not normal (*p* < 0.05). Post-hoc analysis of multiple pairwise comparisons was conducted using the paired Wilcoxon signed-rank test. *p*-values were adjusted using the Bonferroni multiple testing correction method. In the case of the cardiac variables, the normality of the data distribution was not rejected (*p* > 0.05), hence multivariate analysis of variance (MANOVA) for repeated measures was used. The significance of differences between means was determined with Tukey’s test. A minimum level of significance was accepted at α = 0.05.

## 3. Results

As presented in Table 2, the effect of experimental conditions (experimental phase and test) was statistically significant (*p* < 0.05) for the traits studied in addition to rest standing, drowsiness and walk. 

**Figure 1:**
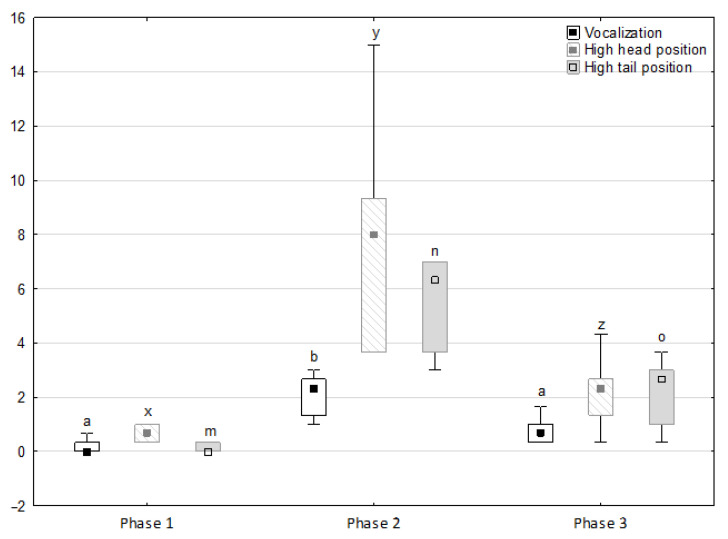
Distribution of vocalisation, high head position and high tail position rates regarding the phase of the experiment (squares show medians, boxes show lower and upper quartiles and whiskers show minimum and maximum values). Statistically significant differences (*p* < 0.05) between medians are marked with different letters: a, b—vocalisation; x, y, z—high head position; m, n, o—high tail position.

**Figure 2:**
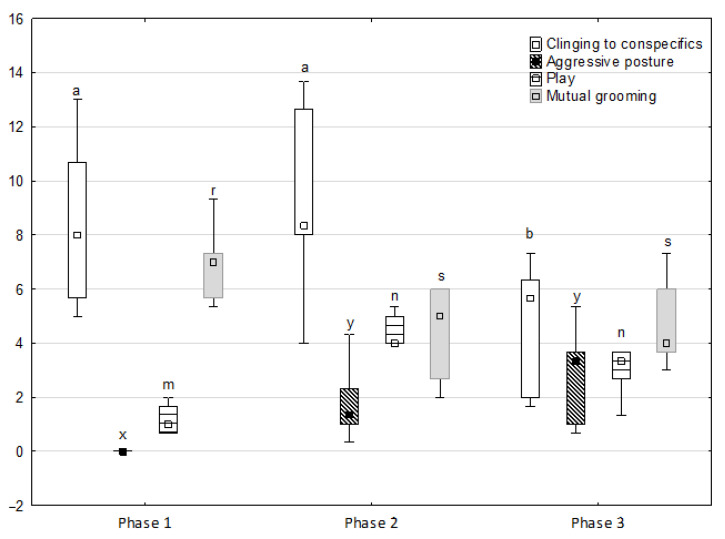
Distribution of clinging to conspecifics, aggressive posture, play and mutual grooming rates regarding the phase of the experiment (squares show medians, boxes show lower and upper quartiles and whiskers show minimum and maximum values). Statistically significant differences (*p* < 0.05) between medians are marked with different letters: a, b —clinging to conspecifics; x, y —aggressive posture; m, n—play; r, s—mutual grooming.

**Figure 3:**
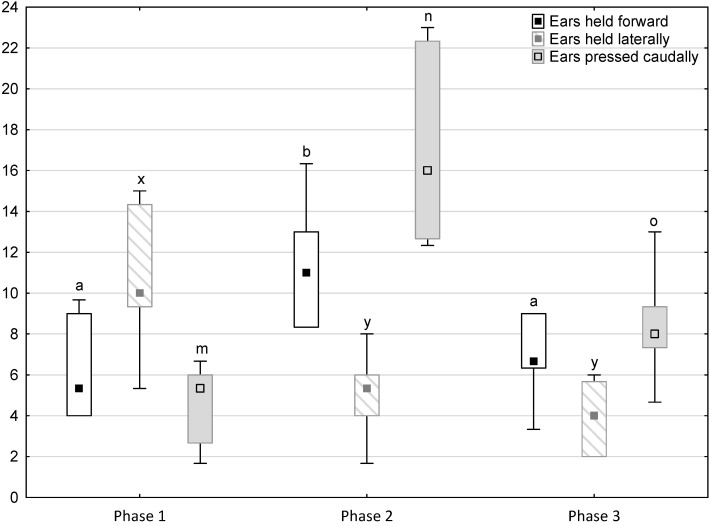
Distribution of ears held forward, ears held laterally and ears pressed caudally rates regarding the phase of the experiment (squares show medians, boxes show lower and upper quartiles and whiskers show minimum and maximum values). Statistically significant differences (*p* < 0.05) between medians are marked with different letters: a, b—ears held forward; x, y—ears held laterally; m, n, o—ears pressed caudally.

**Figure 4:**
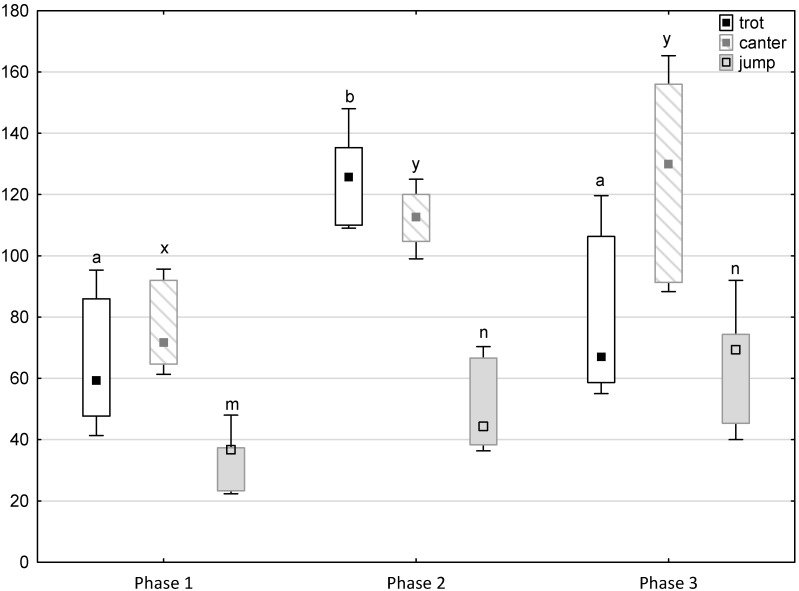
Distribution of trot, canter and jump times regarding the phase of experiment (squares show medians, boxes show lower and upper quartiles and whiskers show minimum and maximum values). Statistically significant differences (*p* < 0.05) between medians are marked with different letters: a, b—trot; x, y —canter; m, n—jump.

**Figure 5:**
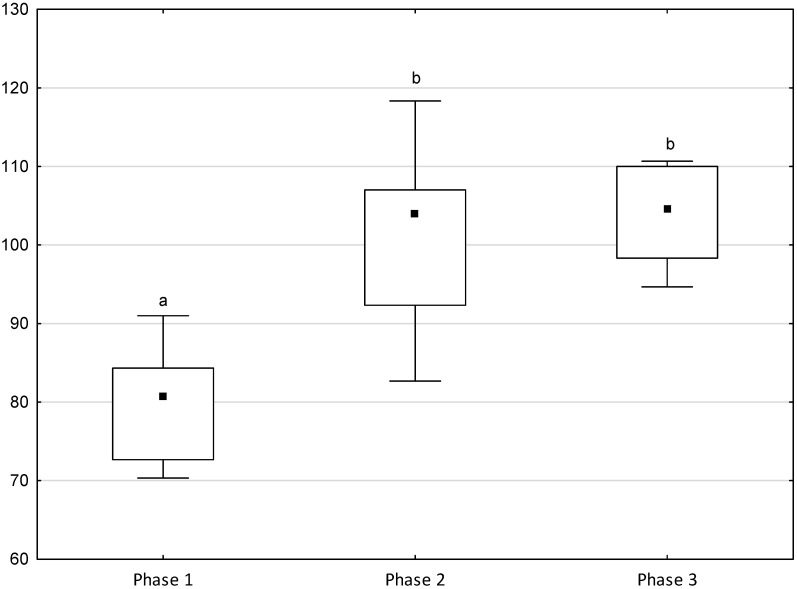
Distribution of the HR regarding the phase of the experiment (squares show means, boxes show lower and upper quartiles and whiskers show minimum and maximum values). Statistically significant differences (*p* < 0.05) between means are marked with different letters: a, b.

Figure 1, Figure 2, Figure 3, Figure 4 and Figure 5 illustrate the answer to the first part of the hypothesis, i.e., whether there were significant differences in the behavioural, locomotor and cardiac traits in the remaining herd (excluding the separated mares) between successive phases of the experiment, regardless of the kind of test. Only those traits that showed statistically significant differences (*p* < 0.05) between medians in the non-parametric test or means in the parametric test were considered. The variables increased in 78.6% of these traits (HR in those) in phase 2 compared to phase 1. They were similar considering clinging to conspecifics and lowered in the case of mutual grooming and ears held laterally. When comparing phase 3 to phase 2, an increase in variables was not observed, whereby the variables remained at a similar level in half of the traits (HR in those) and lowered in another half (vocalisation, high head position, high tail position, clinging to conspecifics, ears held forward, ears pressed caudally and trot).

**Figure 6:**
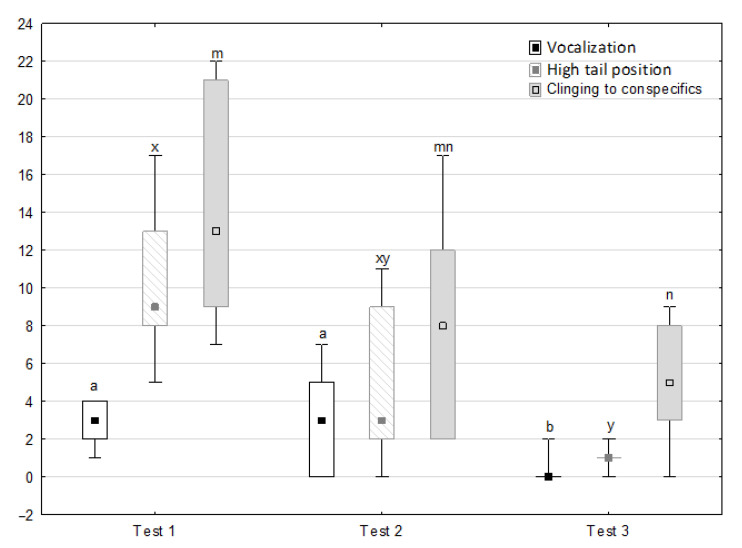
Distribution of vocalisation, high tail position and clinging to conspecifics rates during phase 2 in successive days of the experiment (squares show medians, boxes show lower and upper quartiles and whiskers show minimum and maximum values). Statistically significant differences (*p* < 0.05) between medians are marked with different letters: a, b—vocalisation; x, y—high tail position; m, n—clinging to conspecifics.

**Figure 7:**
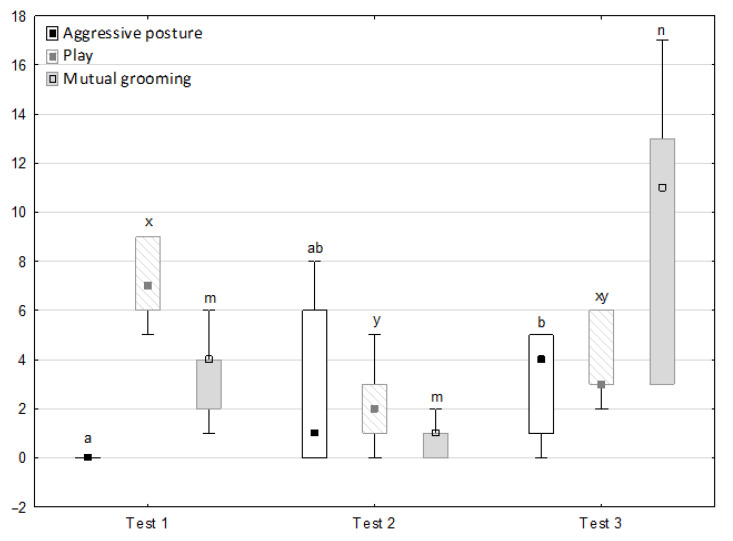
Distribution of aggressive posture, play and mutual grooming rates during phase 2 in successive days of the experiment (squares show medians, boxes lower and upper quartiles and whiskers show minimum and maximum values). Statistically significant differences (*p* < 0.05) between medians are marked with different letters: a, b—aggressive posture; x, y—play; m, n—mutual grooming.

**Figure 8:**
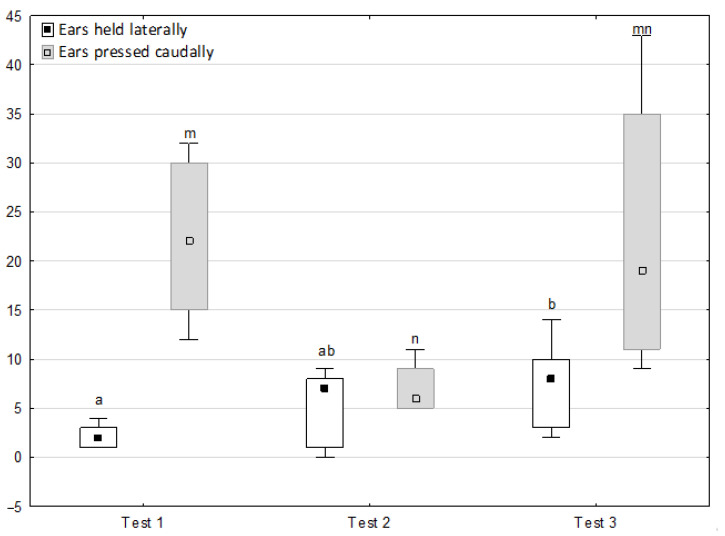
Distribution of ears held laterally and ears pressed caudally rates during phase 2 in successive days of the experiment (squares show medians, boxes show lower and upper quartiles and whiskers show minimum and maximum values). Statistically significant differences (*p* < 0.05) between medians are marked with different letters: a, b—ears held laterally; m, n—ears pressed caudally.

**Figure 9:**
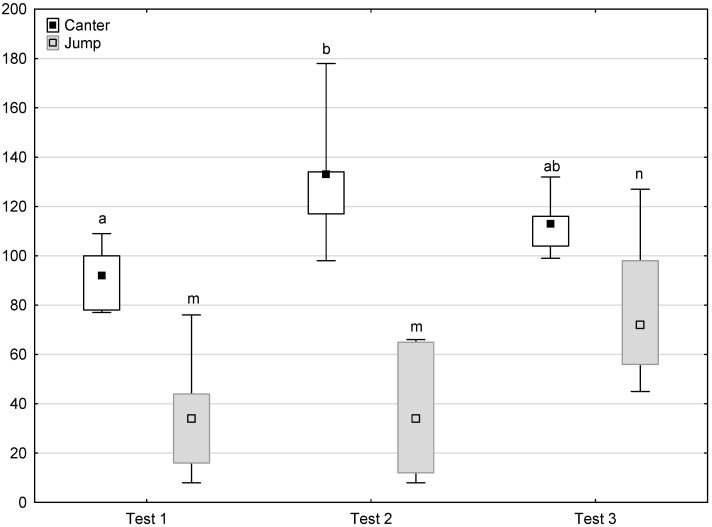
Distribution of canter and jump times during phase 2 in successive days of the experiment (squares show medians, boxes show lower and upper quartiles and whiskers show minimum and maximum values). Statistically significant differences (*p* < 0.05) between medians are marked with different letters: a, b—canter; m, n—jump.

Figure 6, Figure 7, Figure 8 and Figure 9 illustrate the answer to the second part of the hypothesis, i.e., whether the response of the horses included in the remaining herd was different in consecutive tests when differently ranked mares in the herd dominance hierarchy were separated during the crucial phase 2. Only those ten traits that showed statistically significant differences (*p* < 0.05) between medians in phase 2 were considered, irrespective of the differences in phases 1 and 3. Hence, the defecation/urination, high head position, ears held forward, trot, single steps, HR and RMSSD, which did not show significant differences within phase 2, were not considered. The variables were lower in the second test compared to the first test in the case of play and ears pressed caudally, similar considering vocalisation, high tail position, clinging to conspecifics, aggressive posture, mutual grooming, ears held laterally and jump and higher for canter. The variables in the third test were similar in 70.0% cases, lower for the vocalisation and higher for mutual grooming and jump compared to the second test. The variables for play, ears pressed caudally and canter did not differ in the first and third tests, whereas those for vocalisation, high tail position and clinging to conspecifics were lower in the third test compared to the first test and higher for aggressive posture, mutual grooming, ears held laterally and jump.

## 4. Discussion

The results of the analysis show that the social herd was not indifferent to the separation of some individuals. The remaining horses noticed the incident and expressed their agitation with various behaviours, locomotion and changes in nervous system activity. The emotional state of the mares in the remaining herd was manifested by a higher rate of many behaviours: Vocalisation, high head and tail position, aggressive posture, play, ears held forward or pressed caudally, as well as prolonged trot, canter, jumps and an elevated HR. These behaviours, apart from play, are typical for increased vigilance and agitation [25,27]. According to McDonnell and Poulin [29], play occurs mostly in foals and young horses, whereas in adults, it is less frequent in mares than in stallions. It consists of activities having no immediate use or function to the animal but involves pleasure and surprise. Hence, it is not connected with anxiety and may show that the mares studied were not agitated. However, all other behaviours indicate an increase in emotional arousal in the herd in response to the incident. Exemplarily, ears were held forward or pressed caudally more often and held laterally more rarely in response to the separation. According to Hall et al. [26], ears pointing forwards are a sign of interest, alertness and attention, whereas ears pinned back reflect a negative affective state. Many authors describe that the act of the ears being pressed back is associated with agonistic behaviours while the ears being held laterally displays a relaxed horse (e.g., [12,23]). Hence, the increase in the rate of the ears held forward or pressed caudally and the decrease in the rate of the ears held laterally display an elevated emotional agitation in the remaining herd. The decrease in the rate of mutual grooming also corresponds with higher emotional arousal [4,13]. Hartmann et al. [22] indicate neighing, snorting, defecation, increased locomotion, pawing and taking a vigilant posture as typical behaviours associated with the separation of an individual; however, they do not describe behaviours of the remaining group. The authors emphasise that the reaction of the separated individuals varied considerably.

The HR and RMSSD show changes in the sympatho-vagal balance of the nervous system activity. An increased HR shows a shift towards the sympathetic nervous system, whereas elevated RMSSD indicates an opposite shift towards the parasympathetic system [30,31]. An increase in the sympathetic component accelerates the HR, whereas an increase in the vagal tone decelerates it and enhances the RMSSD. The predominance of the sympathetic nervous system activity over the antagonistic parasympathetic system indicates that the mares in the herd sensed the separation of some of them as a stressful situation. The RMSSD, although significantly affected by the separation, did not show any important differences between experimental phases. This suggests that the parasympathetic nervous system is less involved in the horse responses studied than the sympathetic nervous system. It is known that the RMSSD changes, for example, in response to relaxation treatment [32]. Despite the non-significant post hoc differences in the RMSSD, the significant changes in many behavioural, locomotor and HR variables in the social herd, which occurred in response to the short separation of some mares, confirm the first part of our hypothesis.

The return of the individuals after the short separation elicited a less distinct response in the herd, since the rate of behaviours reflecting the emotional arousal decreased or remained at a similar level in comparison to the phase of separation. The behaviours reflecting calmness (play, mutual grooming and ears held laterally) also remained at a similar level to that during separation. It seems that the return of the separated mares began to calm down the herd, which sensed this state as restoring normality. 

The differences in the variables between the tests reveal that the composition of the group of separated mares, which differed in the dominance hierarchy, influenced the response, however inconsistently. Comparing the effect of the separation of the mixed group (second test) to the dominant group (first test) on behaviours connected to agitation, only the time of canter was higher, whereas mutual grooming and ears held laterally were at a similar level and that of play was lower within the rates of behaviours showing calmness. When comparing the separation of the submissive group (third test) with the mixed group, the response of the herd did not consistently tend towards agitation or calmness: The increased jump time displayed when the submissive group was separated indicates a predominance of agitation but decreased vocalisation in accordance with increased mutual grooming shows a predominance of calmness. In turn, the separation of the submissive group compared to the dominant group evoked the increased rate of aggressive posture and jump time, which indicates higher emotional arousal, although simultaneously elevated rates of mutual grooming and ears held laterally as well as decreased vocalisation, high tail position and clinging to conspecifics show a tendency for calmness. 

The separation turned out to be stressful for the remaining herd despite the fact that the horses might have been habituated to such a procedure within everyday routine. As mentioned in the Introduction, separations are frequent in horse management. Hence, the distinct increase in emotional arousal shows how humans’ activities may be important for the horses’ welfare. Opposite to evident changes towards emotional arousal elicited by the separation, the response to the separation of conspecifics variously ranked in the dominance hierarchy was not homogenous. Interestingly, whether a dominant, mixed or submissive group of mares was separated did not affect the remaining herd. It does not seem that the separations carried out according to the experimental design once every four days were anything different than usual management for the mares and, thus, that the order of separation could influence the results. The level of agitation was not differentiated, i.e., the changes did not differ consistently depending on the rank order of the individuals separated. Thus, the results of the study do not provide evidence to support the second part of the hypothesis. It may be suggested that the mares in the remaining herd were indifferent to which mares were separated. The dominance relationships could not have been changed in the herd for such a short period (10 min). Perhaps the dominant mare/mares are needed for other animals in the herd in the case of, for example, a strong alert or protection against a real threat. Presumably, the procedure of separation in our study did not evoke a sufficiently strong alert to make the remaining mares look for a leader’s support. According to Hartmann et al. [22], the main value of horses and other species living in groups is the possibility of mutual vigilance and protection against predators. These authors also observed that there was no effect of social rank of a target horse that was being followed by the remaining group members and suggested that the consequences of removing either higher or lower ranked horses from their group may be overestimated by horse owners. Our results document, with a number of variables, that the rank order of separated mares is not a decisive factor for the response displayed by the remaining herd.

As was mentioned in the Introduction, studies on the horses’ responses to the separation of some individuals from a herd are lacking. Jørgensen et al. [20] undertook this issue but in the context of interaction with humans when a horse was removed from its social group. Hartmann et al. [22] suggested an effect of the proportion of the group that was being removed on whether or not other horses followed the separated individuals. Our experiment was carried out in a small group of mares; however, the group was not much bigger than natural herds regarding the number of mares that develop the dominance hierarchy. Grouping mares with geldings in social herds does not interfere with usual horse behaviour [14], hence it seems that the results may be extrapolated to such groups of different genders. Summing up, humans may be unaware of how their activities affect the emotional arousal of horses. Such activities do not seem to be important, contrary to the actual influence on horses. The cognition of these phenomena may be key to understanding some unclear horse behaviours. Our results indicate horse owners should take into account agitation in the social herd during the separation of some of its members and provide peaceful conditions for such a procedure, whereas the rank of separated individuals does not have to be regarded. The herd, even created by humans, preserves the sensitivity to a temporary loss of its members. In spite of the fact that such situations are constantly repeated in practice, they are stressful for the horses. Inconsistent response to the separation of differently ranked members in the dominance hierarchy may mean that both dominant and submissive members of the herd fulfil their roles in the hierarchy and a lack of them deranges the composition of the herd.

## 5. Conclusions

The results of the experiment reveal evident changes towards emotional arousal in the social herd elicited by a short separation of some conspecifics. The agitation in the remaining herd is elevated after the separation of some conspecifics. The response of the remaining herd is not consistently connected to the composition of the mares separated regarding their rank in the dominance hierarchy.

## Figures and Tables

**Table 1:** Description of the behaviours recorded in the study.

Behaviour	Description
Vocalisation	Signals produced by the nostrils during expiration (snorts, snores and blows).
Defecation/Urination	Elimination of faeces or urine.
High head position	Neck raised over 45 degrees to the horizontal level.
High tail position	Fleshy part of tail outstretched horizontally or elevated above horizontal.
Clinging to conspecifics	Body contact not reciprocated by the receiver. The horse is difficult to split up, inseparable from conspecifics.
Aggressive posture	Ears pressed caudally against the head and neck; head or kick threat.
Rest standing	Standing inactive in a relaxed posture, usually with head slightly lowered. The horse is standing on three or four limbs and can reposition them slightly remaining close to the original position. The head and neck can move without movement of the limbs.
Drowsiness	The horse is standing on three or four limbs without moving in any direction. Ears rotate laterally.
Play	Behavioural elements and sequences similar to serious adult fighting behaviour but of a play character performed while in motion at any gait. Play is directed at another individual, which may or may not reciprocate.
Mutual grooming	Grooming by gentle nipping, nuzzling or rubbing involving the muzzle and teeth while standing beside one another, usually head to shoulder or head to tail.
Position of ears	Ears held forward: Curiosity, alert Ears held laterally: Relaxation, drowsiness, boredom Ears pressed caudally: Aggression, frustration

**Table 2:** The effect of experimental conditions (experimental phase and test) on behavioural (Friedman test probability value) and cardiac activity traits (MANOVA test probability value).

Behavioural Traits	*p*-Value	Locomotor Traits	*p*-Value
Vocalisation	0.0020 *	Walk	0.0716
Defecation/Urination	0.0344 *	Trot	0.0029 *
High head position	0.0001 *	Canter	0.0022 *
High tail position	0.0002 *	Jumps	0.0210 *
Clinging to conspecifics	0.0068 *	Single steps	0.0274 *
Aggressive posture	0.0114 *	**Cardiac Activity Traits**	***p*-Value**
Rest standing	0.3801	HR	0.0002 *
Drowsiness	0.1740	RMSSD	0.0002 *
Play	0.0002 *		
Mutual grooming	0.0011 *		
Ears held forward	0.0205 *		
Ears held laterally	0.0003 *		
Ears pressed caudally	0.0015 *		

* *p* < 0.05.

## Data Availability

The data presented in this study are available on request from the corresponding author.
